# Evaluation of metabolic equivalents of task (METs) in the preoperative assessment in aortic repair

**DOI:** 10.1186/s12893-021-01143-0

**Published:** 2021-03-13

**Authors:** Alicja Zientara, Igor Schwegler, Omer Dzemali, Hans Bruijnen, Alain Bernheim, Florian Dick, Nicolas Attigah

**Affiliations:** 1 Department of Cardiac Surgery, Royal Brompton and Harefield Hospital, Sydney Street, London, SW3 6NP UK; 2 Department of Vascular Surgery, Triemli Hospital, Birmensdorferstrasse 496, 8063 Zürich, Switzerland; 3 Department of Cardiac Surgery, Triemli Hospital, Birmensdorferstrasse 496, 8063 Zürich, Switzerland; 4 Department of Vascular and Thoracic Surgery, Augsburg Hospital, Stenglinstr. 2, 86156 Augsburg, Germany; 5 Department of Cardiology, Triemli Hospital, Birmensdorferstrasse 496, 8063 Zürich, Switzerland; 6 Department of Vascular Surgery, Cantonal Hospital, Rohrschacher Strasse 95, 9007 St. Gallen, Switzerland

**Keywords:** Metabolic equivalent of task (MET), Aortic repair, Preoperative assessment, Functional capacity

## Abstract

**Background:**

Reliable prediction of the preoperative risk is of crucial importance for patients undergoing aortic repair. In this retrospective cohort study, we evaluated the metabolic equivalent of task (MET) in the preoperative risk assessment with clinical outcome in a cohort of consecutive patients.

**Methods:**

Retrospective analysis of prospectively collected data in a single center unit of 296 patients undergoing open or endovascular aortic repair from 2009 to 2016. The patients were divided into four anatomic main groups (infrarenal (endo: n = 94; open: n = 88), juxta- and para-renal (open n = 84), thoraco-abdominal (open n = 13) and thoracic (endo: n = 11; open: n = 6). Out of these, 276 patients had a preoperative statement of their functional capacity in metabolic units and were evaluated concerning their postoperative outcome including survival, in-hospital mortality, postoperative complications, myocardial infarction and stroke, and the need of later cardiovascular interventions.

**Results:**

The median follow-up of the cohort was 10.8 months. Patients with < 4MET had a higher incidence of diabetes mellitus (p = 0.0002), peripheral arterial disease (p < 0.0001), history of smoking (p = 0.003), obesity (p = 0.03) and chronic obstructive pulmonary disease (p = 0.05). Overall in-hospital mortality was 4.4% (13 patients). There was no significant difference in the survival between patients with a functional capacity of more than 4 MET (220 patients, mean survival: 74.5 months) and patients with less than 4 MET (56 patients, mean survival: 65.4 months) (p = 0.64). The mean survival of the infrarenal cohort (n = 169) was 74.3 months with no significant differences between both MET groups (> 4 MET: 131 patients, mean survival 75.5 months; < 4 MET: 38 patients, mean survival 63.6 months. p = 0.35). The subgroup after open surgical technique with less than 4 MET had the lowest mean survival of 38.8 months. In 46 patients with > 4MET (20.9%) perioperative complications occurred compared to the group with < 4MET with 18 patients (32.1%) (p = 0.075). There were no significant differences in both groups in the late cardiovascular interventions (p = 0.91) and major events including stroke and myocardial infarction (p = 0.4) monitored during the follow up period. The risk to miss a potential need for cardiac optimization in patients > 4MET was 7%.

**Conclusion:**

The functional preoperative evaluation by MET in patients undergoing aortic surgery is a useful surrogate marker of perioperative performance but cannot be seen as a substitute for preoperative cardiopulmonary testing in selected individuals. *Trial registration* clinicaltrials.gov, registration number NCT03617601 (retrospectively registered).

## Introduction

For several reasons a reliable preoperative risk evaluation is of particular importance in vascular patients. Foremost the majority of indications is made for patients in sixth to eighth decade of their lives and a large portion of it remains of preventive intention such as elective aneurysm repair or carotid surgery for asymptomatic occlusive disease. Furthermore the presence of atherosclerotic disease is supposed to bear an own increased perioperative risk [1, 2]. Almost 40% of all perioperative complications are made up by cardiac incidences [3]. In high-risk patients undergoing non-cardiac operations the 30-day mortality for cardiovascular death or myocardial infarction has been estimated to be over 5% [4].

A prophylactic coronary revascularization in patients with stable coronary artery disease by percutaneous coronary intervention (PCI) or bypass operation did not show any benefit concerning the reduction of mortality or occurrence of perioperative myocardial infarction when compared to medical treatment [5, 6].

The preoperative assessment of the metabolic equivalent of task (MET) is an easy clinical evaluation of functional capacity or exercise tolerance of an individual. A MET is defined as the resting metabolic rate, that is the amount of the consumed oxygen at rest (approximately 3.5 ml = 0_2_/kg/min i.e. 1.2 kcal/min for a 70 kg person) [7]. According to the MET concept a patient would be considered as “fit for surgery” when the stairs of two flights can be climbed and the housework can be fully managed by oneself. Preoperative assessment of MET also is used for the evaluation of the perioperative risk for cardiac complications.

The current guidelines of the European Society of Cardiology and European Society of Angiology (ESC/ESA) restrain from preoperative cardiac testing in non-cardiac surgery as it has failed to improve perioperative outcome [8]. Alike, the current guidelines of the American College of Cardiology and American Heart Association (ACC/AHA) from 2014 recommend no further cardiac testing in patients undergoing non-cardiac surgery with moderate risk for a major cardiac event (MACE) with a functional capacity that exceeds 4 MET with class IIb evidence [9]. Although these guidelines are widely supported and implemented, they are still not well validated for specific vascular interventions i.e. aortic operations.

In this retrospective cohort study we investigated the MET concept in the preoperative risk assessment with clinical outcome parameters in a cohort of consecutive patients who received open or endovascular abdominal, thoraco-abdominal or thoracic aortic repair. We hypothesize that patients with a preoperative status of 4 MET and more had less perioperative complications, suffered less from postoperative myocardial infarction and stroke and had a lower in-hospital mortality compared to patients with a status under 4 MET. This might support the recommendation of non-indicated cardiac testing in patients who are fit for surgery.

## Methods

### Patients and data collection

All relevant patients’ data were retrospectively extracted from the Swiss Vasc Registry, a prospective, mandatory nationwide computer-based vascular registry in Switzerland. After the extraction of the center-associated clinical data all files were completed with the current patients’ follow-up and rechecked for obvious entry mistakes in the hospital software Medfolio. From May 2009 till March 2016, 296 patients underwent open or endovascular aortic repair and were divided into four main groups depending on infrarenal, juxta- and para-renal, thoraco-abdominal and thoracic pathology (Table 1). For further analysis, 20 patients who underwent an emergency operation without known MET status were excluded. Beside demographic parameters, type of operation and urgency, co-morbidities and cardiovascular risk factors were documented. Perioperative data included postoperative complications and reoperations, myocardial infarction and stroke, in-hospital mortality, and the need of later cardiovascular interventions.

**Table 1:** Distribution of the four aortic intervention groups

Morphology of the four groups	Open repair	Endovascular	n
Infrarenal	88	94	182
Juxta-/pararenal	84	0	84
Thoraco-abdominal	13	0	13
Thoracic	6	11	17
Total number			296

The study was approved by the Cantonal Ethics Committee of Zurich under the Protocol Number 2017-00801 in June 2017 and adheres to the principles of reported research on human beings set forth in the Helsinki Declaration. The study has been registered under clinicaltrials.gov with the Registration Number NCT03617601.

### Preoperative assessment

Before 2009, all patients underwent routinely a preoperative cardiac assessment regardless of their functional capacity or planned operation. Since 2009, MET concept was gradually adopted and more patients with > 4MET went for open or endovascular aortic repair without preoperative testing. Before 2014, the majority of patients underwent preoperative cardiac assessment. After the ACC/AHA recommendation of 2014, patients with a capacity > 4MET and without pathologic cardiac history were consequently operated without preoperative assessment [9]. In case of doubt that the patients’ information was reliable, MET testing was done during consultation by the operating surgeon by completing the anamnesis and performing a stair climbing test. Because of the increased circulatory stress and the large extend of this procedure, all patients receiving thoraco-abdominal aortic repair underwent cardiac and pulmonary check-up routinely at any time. The routine cardiac check-up included electrocardiogram (ECG), chest x-ray and dobutamine stress echocardiography (DSE) [10]. The pulmonary evaluation included body plethysmography and in case of major diffusion capacity reduction a cardio-pulmonary exercise testing (CPET). According to our institutional protocol every patient with atherosclerotic disease received single anti-aggregative treatment usually by Aspirin 100 mg p.o. once daily as secondary prophylaxis for cardiovascular events and a statin therapy, if not yet established previously. Furthermore, during preoperative anesthesiologic assessment antihypertensive therapy was established, if needed. All patients undergoing aneurysm repair received a carotid duplex to rule out concomitant hemodynamically significant carotid stenosis in order to minimize perioperative risk for stroke.

### Operative technique in open infra-, juxta-, and thoraco-abdominal repair

All infrarenal aneurysms received preferably balloon occlusion of the iliac arteries in order to avoid clamping damage. An aneurysm was labeled juxtarenal, if at least one renal artery had to be clamped suprarenal in order to perform the proximal anastomosis. If there was suprarenal clamping of both renal arteries, renal cold perfusion was established whenever possible. In case of thoraco-abdominal aortic repair patients were positioned in a right lateral decubitus position to facilitate aortic exposure via thoraco-phrenico-lumbotomy. Airway management was achieved with a double lumen endotracheal tube to allow unilateral right lung ventilation. Operation was performed with partial left heart bypass with suprainguinal cannulation of the external iliac arty and vein without hypothermia [11]. All patients received spinal cord monitoring with sensory evoked potentials (SEPs) and motor evoked potentials (MEPs) and prophylactic spinal cord drainage and pressure monitoring prior to surgery [12].

### Operative strategy in infrarenal and thoracic endovascular repair

All patients receiving endovascular repair were operated with a femoral cut-down. Indication for endovascular repair was within the instructions for use (IFU) of the chosen endoprosthesis. Endovascular repair of aortic pathologies in landing zone < 3 according to Mitchell and Ishimaru were operated in functional cardiac arrest with rapid pacing [13]. If treatment length exceeded 20 cm and required deployment in landing zone 2, or in the presence of prior aortic surgery, respectively carotid-subclavian bypass was carried out prior to endovascular repair in order to minimize the risk of spinal cord malperfusion [14].

### Follow-up of patients

All patients with open infrarenal aortic repair had a duplex scan from the referring angiologist 3 months postoperative and clinical evaluation by the operating surgeon in order to evaluate clinical status and rule out incisional hernia. From then on, ultrasound and a physical examination was done every year. Patients with thoraco-abdominal open repair received an Angio-CT scan 3 months after surgery and then were followed with ultrasound. All endovascular repairs underwent postoperative CT scan after 3 months and one year. If sac diameter was in regression and endoleaks were absent it was switched to an ultrasound or contrast enhanced duplex scan.

### Statistical methods

Descriptive statistics were generated by counting and calculation of percentages of the nominal and ordinal variables. Numeric variables were described with mean and standard deviation. Survival analysis was done according to the method described by Kaplan and Meier. For the analysis of differences between the groups the log rank test was used. Twenty patients out of 296 were emergencies without any information about preoperative MET status. These patients were excluded from the comparison between patients with > 4MET and < 4MET according demographic data, survival and follow up. Perioperative complications, postoperative cardiac interventions and postoperative severe events (stroke, myocardial infarction) were calculated separately for the emergency group without MET status. In-hospital mortality was calculated for all patient regardless of their MET status focusing on the localization of the procedure (infrarenal, juxtarenal, thoraco-abdominal, thoracic). Statsdirect software (Version 2.7.3, Statsdirect Ltd, Cheshire, UK) was used for all statistical analyses.

### Sensitivity/specificity analysis

Based on additional cardiac assessment we were able to calculate the sensitivity and specifity of the diagnostic validity of MET. Sensitivity being the proportion of patients with a pathologic result of the assessment among patients with < 4MET, specificity the proportion with a normal result among patients with > 4MET.

### Sample size calculation

Based on results of the study—power of the results of the retrospective risk analysis—we performed a sample size calculation. This gives us an indication of the number of patients needed to detect a statistically significant difference, assuming that our hypothesis is true. Power: 100%—probability of not detecting the “true” difference. For this calculation we set the—error at 20%, power therefore being 80%.

## Results

### Demographic parameters of patients with > 4MET and < 4MET

Patients with < 4MET had a significant higher incidence of diabetes mellitus (p = 0.0002), peripheral artery occlusive disease (p < 0.0001), history of smoking (p = 0.003), adipositas (p = 0.03) and chronic obstructive pulmonary disease (p = 0.05). Furthermore, this group had a tendency to more previous cardiovascular interventions, otherwise the two cohorts were comparable (Table 2)*.*

**Table 2:** Demographic parameters

Demographic parameters	METS		p
	> 4 (n = 220)	< 4 (n = 56)	
Age (median) (Interquartile range)	71.8 (65.0 – 77.3)	71.0 (64.5 – 76.5)	0.80
Gender (female)	30 (13.6%)	13 (23.2%)	0.09
Localisation			0.21
Infrarenal	131 (59.6%)	38 (67.9%)	
Juxtarenal	61 (27.7%)	16 (28.6%)	
Thoracoabdominal	13 (5.9%)	0 (0%)	
Thoracic	15 (6.8%)	2 (3.6%)	
Nicotine	125 (56.8%)	44 (78.6%)	0.003
Arterial hypertension	145 (65.9%)	39 (69.6%)	0.75
Diabetes (*on insuline)	13 (5.9%); (*6 (2.7%))	11 (19.6%); (*6 (10.7%))	0.0002
Adipositas	31 (14.1%)	31 (55.4%)	0.03
Dyslipidemia	105 (47.7%)	15 (26.8%)	0.37
Coronary artery disease	69 (31.4%)	19 (33.9%)	0.75
COPD (chronic obstructive pulmonary disease)	43 (19.5%)	18 (32.1%)	0.05
Renal insufficiency	24 (10.9%)	9 (16.1%)	0.36
Stroke	19 (8.6%)	6 (10.7%)	0.53
Myocardial infarction	22 (10%)	10 (17.9%)	0.11
Peripheral arterial occlusive disease	38 (17.3%)	25 (44.6%)	< 0.0001
Previous cardiovascular interventions* (*various procedures in one patient possible)	72 (32.7%)	23(41.1%)	0.24
Aortocoronary bypass operation	28 (12.7%)	8 (14.3%)	0.76
Coronary stenting	27 (12.3%)	7 (12.5%)	0.96
Valve operation	12 (5.5%)	2 (3.6%)	0.57
Aortic operation/procedure	8 (3.6%)	5 (8.9%)	0.09
Peripheral vascular operation	6 (2.7%)	4 (7.1%)	0.11
Emergency / urgent procedures	17 (7.7%) / 15 (6.8%)	3 (5.4%) / 5 (8.9%)	0.54/ 0.59

Excluded: 20 patients with unclear METS status; 19 emergencies, 1 urgent operation

### Follow up and survival

**Fig. 1:**
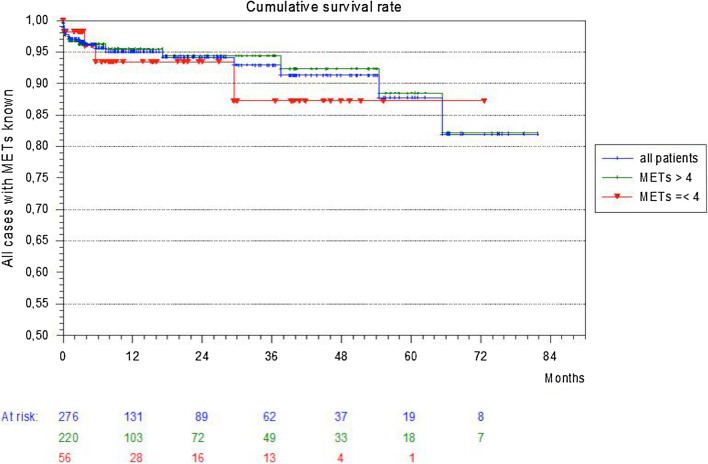
The Kaplan Meier survival curve of the whole cohort subdivided in patients with preoperative status of > 4 MET and < 4 MET

**Fig. 2:**
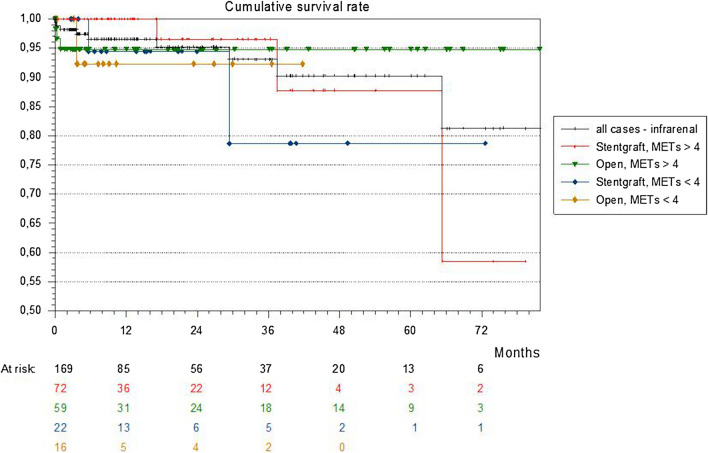
The Kaplan Meier survival curve after infrarenal aortic procedure; all four subgroups (open vs endovascular, > 4MET vs < 4MET) show no significant differences concerning the mean survival (p = 0.82)

The median follow-up of the patients’ cohort was 10.8 months. Mean survival of the whole cohort with recorded MET status (n = 276) was 74 months. There was no significant difference between patients with a functional capacity of more than 4 MET (220 patients, mean survival: 74.5 months) and patients with less than 4 MET (56 patients, mean survival: 65.4 months) (p = 0.64) (Fig. 1). The mean survival of the infrarenal cohort (n = 169) was 74.3 months with no significant differences between both MET groups (> 4 MET: 131 patients, mean survival 75.5 months; < 4MET: 38 patients, mean survival 63.6 months. p = 0.35). The infrarenal cohort was subdivided in four groups based on the operative technique (open or endovascular) and the preoperative MET status (> 4 or < 4 MET). The Kaplan Meier survival of all four subgroups shows no significant differences concerning the mean survival (p = 0.82) (Fig. 2). The subgroup after open surgical technique with less than 4 MET had the lowest mean survival of 38.8 months.

### Perioperative complications and reoperations

In 46 patients with > 4METS (20.9%) perioperative complications occurred compared to the group with < 4METS with 18 patients (32.1%). There was no significant difference between both groups (p = 0.075), but a slight tendency for more complications in patients with < 4METS (32.1% vs 20.9%).

Fifty-seven complications occurred in the 46 patients with > 4MET. Of these 57 complications, 21 (36.8%) led to major reoperations and 11 (19.3%) to minor reoperations. Compared to the complications in the patient group with < 4MET, there was no significant difference regarding the number of major and minor reoperations (p = 0.89 major; p = 0.43 minor) (Table 3).

**Table 3:** Perioperative complications

		METS	
	> 4 (n = 220)	< 4 (n = 56)	p
Perioperative complications			
Number of patients with complications	*46 *(*20.9%*)	18 (32.1%)	0.07
All complications (various complications per one patient included)	57 (100%)		
Minor re-operation	11 (19.3%)	7 (26.9%)	0.43
Major re-operation	21 (36.8%)	10 (38.5%)	0.89
No re-operation	25 (43.9%)	9 (34.6%)	0.43

Minor: Limb compartment, Pacemaker implantation, Perm-Cath implantation, Wound revision, Drainage

Major: Abdominal compartment, bleeding with re-laparotomy/re-thoracotomy, graft occlusion, intestinal resection

Further complications: renal insufficiency/dialysis, rhythm, endoleak (without therapy), bronchial infection, delirium

### MET and late postoperative cardiovascular interventions

Sixteen percent of all patients irrespective of their functional capacity underwent a cardiovascular intervention after the initial hospitalization during the follow-up period (36 patients (16.4%) with > 4MET; 9 patients (16.1%) with < 4MET) without significant difference among the groups (p = 0.91).

### MET and major events (stroke or myocardial infarction) after hospitalization

In the group of patients with > 4MET (220 patients), 3.6% (8 patients) developed a myocardial infarction (MI) after hospitalization and 1.8% (4 patients) had a stroke, which results in a cumulative rate of 5.5% (12 patients) with major events after hospitalization. Compared to the group with < 4MET (56 patients), 8.9% (5 patients) suffered a myocardial infarction and no stroke. Although, there was no significant difference among the groups (p = 0.40), there might be a slight tendency to more myocardial infarction in the group with < 4MET.

### MET and in-hospital death

Thirteen patients out of the whole cohort (n = 296) died, which results in an in-hospital mortality of 4.4% for all procedures and approaches (7 patients with > 4MET (3.2%), 2 patients with < 4MET (3.6%), 4 patients with unknown MET after emergency operation (20%). In the infrarenal group of 182 patients the in-hospital mortality was 3.8%. Seven patients died, two from cardiovascular events, five from other events. Six of these seven patients were operated conventionally by laparotomy, one received an endovascular prosthesis. In-hospital mortality after juxtarenal approach (n = 84) was 4.8% (2 cardiovascular deaths, 2 other deaths). During follow up, there were 8 deaths in the infrarenal (3 cardiovascular and 5 other) and 2 deaths (both cardiovascular) in the juxtarenal group.

In the thoraco-abdominal (n = 13) and thoracic approach group (n = 17) respectively, one patient died from cardiovascular cause, which results in an in-hospital mortality of 7.7% and 5.9%. During follow up, there were no further deaths in both groups.

In the subgroup analysis of infrarenal patients there were no significant differences concerning in-hospital mortality with regard to preoperative MET status (p = 0.99).

### Sensitivity of MET status for perioperative cardiovascular risk assessment

**Fig. 3:**
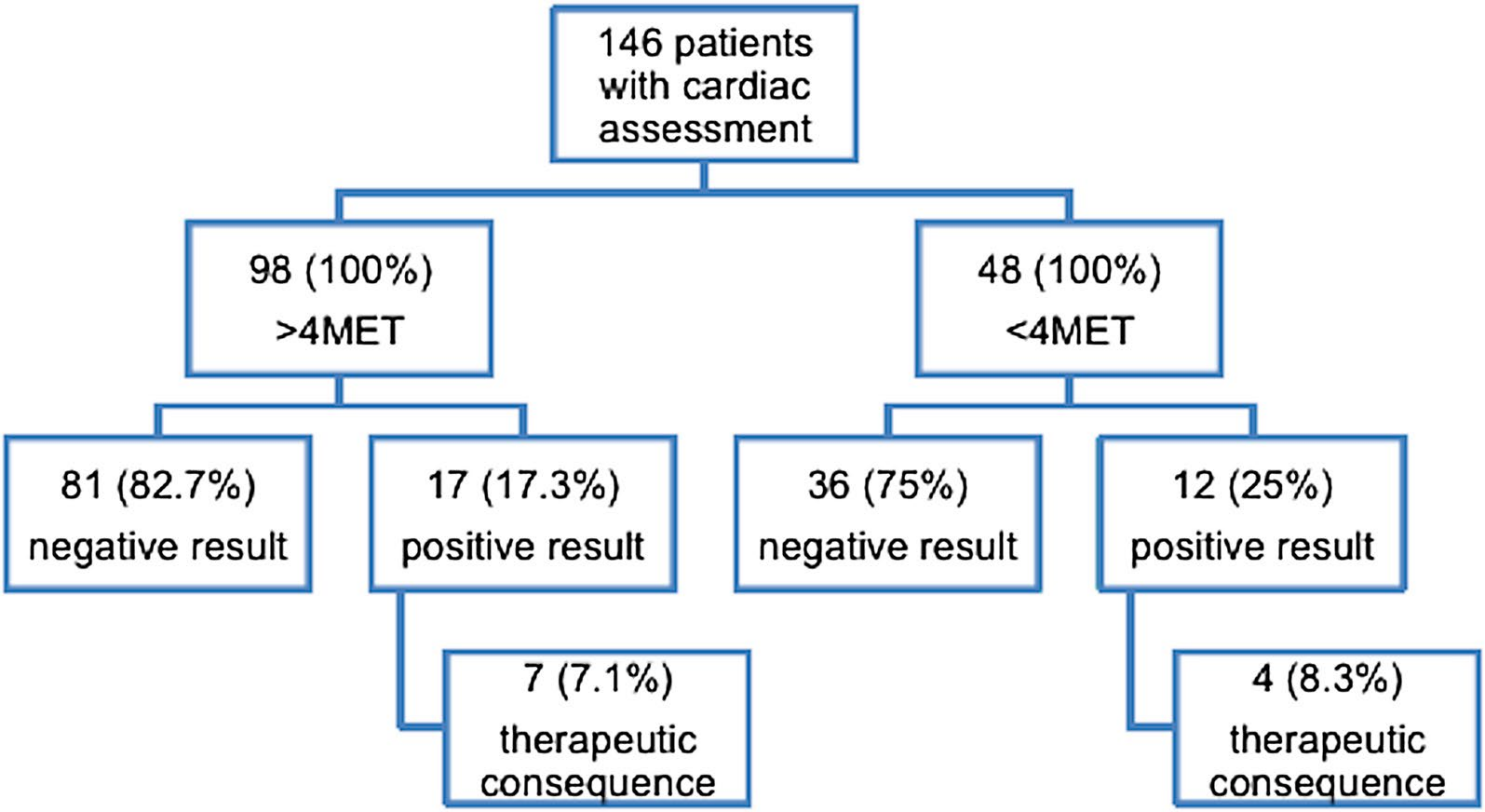
Sensitivity of MET status for perioperative cardiovascular risk assessment: All 148 patients received a preoperative cardiac assessment. The graph underlines the risk of missing a potential need for cardiac optimization in both MET groups.

One hundred patients with > 4MET received concomitant cardiac assessment before the operation. In two of these patients the result of the assessment was unknown, while 81 showed normal results and 17 had pathological findings (= 98 patients with > 4MET and cardiac assessment). In the group with < 4MET, 48 patients underwent preoperative cardiac work-up. Out of these 36 had normal results, whereas 12 showed pathological results in stress echocardiography or coronary angiography. Thus, sensitivity of functional capacity assessment with MET was 41% with a low positive predictive value of 25%. In the group of > 4MET with preoperative cardiac work-up (n = 98), 17 patients showed pathological results. In 7 (7%) out of these this had therapeutic consequences. In the group of < 4MET (n = 48), 4 (8.3%) out of 12 patients received preoperative interventions as a result of abnormal findings. Thus, the risk to miss a potential need for cardiac optimization in patients > 4MET was 7% (Fig. 3).

## Discussion

The aim of this study was to investigate, if the preoperative MET status as a functional and cost-effective parameter may contribute to the avoidance of unnecessary preoperative testing and may identify cardiovascular risk in indicated cases. Since there is no prospective study according to consequent preoperative cardiac evaluation on the basis of MET status, we would like to provide these retrospective data as an initial starting point for further discussion.

In our study, 13 patients died, which resulted in an in-hospital mortality of 4.4% for the whole cohort. In the infrarenal group of 182 patients the in-hospital mortality was 3.8% with no statistical difference between open and endovascular repair. This is consistent with those of major prospective studies investigating infrarenal aortic repair [15, 16]. This study could not demonstrate a statistical difference between patients achieving more or less of a functional capacity of 4 MET in terms of survival and overall complications. The sensitivity of MET assessment in terms of the detecting of pathologic cardiac findings is with 41% quite low. This is most probably due to the flaw of retrospective design and the rather low sample size in this study. Using the figures of the EVAR 2 trial the 30-day mortality in the endovascular group judged unfit for surgery was 7.3%. The patient cohort in EVAR 2 was categorized as unfit for surgery based on their past medical history of myocardial infarction, arrhythmias [17]. Irrespective of the operative method in our cohort the mortality in patients > 4MET was 4.3% and 6.1% in those with < 4MET. A sample size calculation taking into account the given numbers with a hypothesis of a better outcome of the > 4MET patients would claim a patients number of approximately 7300 patients to include into a prospective study, by planning the test in an one-sided design (i.e. > 4MET is associated with lower mortality), 4000 patients would have to be included.

In 1999, Reilly et al. reported that the self-reported exercise tolerance is a valuable tool in order to predict in-hospital perioperative risk [18]. We adopted the MET concept gradually mainly for infrarenal and juxtarenal aortic repair. Apart from cost reduction this regimen is supposed to avoid unnecessary preoperative investigations, which is claimed by current guidelines [8, 19].

The most commonly used, and a simple method to categorize a patients’ operative risk preoperatively is the ASA classification, which is easy to assess and is based on the past medical history and judgement of the assessing physician. This simple categorization correlates astonishingly well with postoperative outcome [19–21]. As cardiovascular morbidity is predominately expected in vascular patients it is not the only potential factor of comorbidity causing postoperative problems. For instance concomitant impairment of liver function in cirrhotic patients or chronic obstructive pulmonary disease (COPD) are independent risk factors for considerably increased postoperative mortality in general major surgery [21–23]. Taking this into account it is clear that the surgeons intuition gives a good estimate of the perioperative risk as there is evidence that the surgeons personal judgment on the patients’ individual risk correlates reliably with postoperative outcome as well [24].

So far there have been only few reports concerning validity of preoperative risk assessment in non-cardiac surgery and none, to our knowledge, in patients undergoing aortic surgery. In patients undergoing thoracic surgery there is evidence that stair climbing capacity is a good predictor of mortality. In patients undergoing high-risk non-cardiac surgery the inability to climb at least two flights of stairs did not provide an increased risk of perioperative mortality but was associated with more cardio-pulmonary complications. However, the fraction was less than 10% [18, 25, 26].

In order to give valid data according to efficacy and safety of the functional status assessment in patients undergoing aortic surgery prospective data in by far larger numbers would be required. However, it seems that even in high-risk operations such as aortic surgery the MET assessment of the patients gives a good estimate of individual physical fitness and overall physiological reserves as our data showed that the group < 4MET had a significant higher percentage of diabetics, claudicants and smokers. Most probably due to small sample sizes our data could not show any difference in mortality or cardiovascular events in patients with more or less than 4 MET in patients undergoing aortic repair. Furthermore, the study is flawed by its retrospective design and the heterogeneity of data concerning operative technique (open or endovascular approach) and the extent of preoperative cardiac testing. However, since there is so far no prospective study evaluating the predictive quality of MET status in aortic surgery, the limited results could encourage the implementation of the MET status in further prospective trials or large registries.

## Conclusion

The concept of MET analysis as a surrogate marker of patients’ performance in the preoperative assessment of patients undergoing aortic surgery does not substitute cardiopulmonary testing in indicated cases as it failed to indicate a clear threshold for extensive preoperative cardiac assessment in those patients.

## Data Availability

The datasets generated and/or analyzed during the current study are not publicly available due to patient privacy and security of electronic medical information but are (anonymized) available from the corresponding author on reasonable request.

## Abbreviations

ACC/AHA: American College of Cardiology/American Heart Association
COPD: Chronic obstructive pulmonary disease
CPET: Cardio-pulmonary exercise testing
CT: Computed tomography
DSE: Dobutamine stress echocardiography
ECG: Electrocardiogram
ESC/ESA: European Society of Cardiology/European Society of Angiology
IFU: Instructions for use
MACE: Major cardiac event
MEPs: Motor evoked potentials
MET: Metabolic equivalent of task
MI: Myocardial infarction
PCI: Percutaneous coronary intervention
SEPs: Sensory evoked potentials
